# Complete mitochondrial genomes of two scaphopod molluscs

**DOI:** 10.1080/23802359.2019.1666689

**Published:** 2019-09-23

**Authors:** Kevin M. Kocot, Tim Wollesen, Rebecca M. Varney, Megan L. Schwartz, Gerhard Steiner, Andreas Wanninger

**Affiliations:** a Department of Biological Sciences, University of Alabama, Tuscaloosa, AL, USA;; b Developmental Biology Unit, European Molecular Biology Laboratory, Heidelberg, Germany;; c School of Interdisciplinary Arts and Sciences, University of Washington, Tacoma, WA, USA;; d Department of Integrative Zoology, University of Vienna, Vienna, Austria

**Keywords:** Scaphopoda, Dentaliida, Gadilida, Mollusca, *Antalis entalis*

## Abstract

Complete mitochondrial genomes were determined for two scaphopod molluscs: the dentaliid *Antalis entalis* and an unidentified Antarctic gadilid. Both genomes are complete except, in Gadilida sp. indet., a short stretch of nad5 was undetermined and trnR could not be annotated. Organization of the Gadilida sp. genome is nearly identical to that previously reported for the gadilid *Siphonodentalium* whereas trnK, nad5, trnD, nad4, and nad4l are transposed to the opposite strand in the previously published *Graptacme* genome relative to that of *Antalis*. Phylogenetic analysis of the 13 protein-coding and 2 rRNA genes recovered Scaphopoda, Gadilida, and Dentaliida monophyletic with maximal support.

Scaphopoda is a clade of burrowing marine molluscs characterized by tubular shells and anterior feeding tentacles called captacula (Reynolds and Steiner 2008). Phylogenomic analyses have shown that Scaphopoda forms a clade with Gastropoda and Bivalvia, but relationships among these three remain unclear (Kocot et al. 2011; Smith et al. 2011). The ∼600 extant species of scaphopods are divided into Dentaliida and Gadilida (e.g., Steiner and Dreyer 2003), but the taxonomy within these groups needs revision. Such work is hindered by limited molecular resources for the group. In particular, just one mitochondrial (mt) genome is available from each of the two major lineages. Here, we present annotated mt genomes for an additional representative of each scaphopod lineage in order to provide additional resources for evolutionary studies of this group.

Adult *Antalis entalis* were collected off Roscoff, France at 25 m. The mt genome of an unidentified species of Gadilida was obtained from an environmental sample collected from the Weddell Sea, Antarctica (75°44′45.132″ S, 31°15′12.708″ W) at 587 m. The shell of the *Antalis* specimen used and a second specimen from the same locality were deposited in the Alabama Museum of Natural History (ALMNH accessions 21272 and 21273, respectively). DNA was extracted using CTAB + phenol/chloroform. For *Antalis*, DNA was sent to the Genomic Services Lab at Hudson Alpha for Illumina PCR-free library preparation and sequencing using ½ lane of a HiSeq X. For Gadilida sp., an Illumina Nextera library was prepared and sent to Macrogen for sequencing on a HiSeq 4000 using ∼1/24 lane.

Assembly was performed with Spades 3.12.0 (Bankevich et al. 2012). This did not yield a complete mt genome for *Antalis*, so Norgal (Al-Nakeeb et al. 2017) was used. Assembled mt genomes were annotated using MITOS 2 (Bernt et al. 2013) with the invertebrate genetic code. The assembled *Antalis* (NCBI MN098312) and Gadilida sp. (NCBI MN104231) mt genomes are 14,836 bp and 13,789 bp, respectively. Both appear complete except, in Gadilida sp., part of nad5 is undetermined and trnR could not be annotated. Aside from differences in the relative positions of trnA, trnH, and possibly trnR, the Gadilida sp. mt genome organization is identical to that of *Siphonodentalium lobatum* (Dreyer and Steiner 2004). Organization of the *Antalis* mt genome was similar to that of *Graptacme eborea* (Boore et al. 2004) except trnK, nad5, trnD, nad4, and nad4l are transposed to the minus strand in *Graptacme* relative to *Antalis*.

The 13 protein-coding and 2 rRNA genes were aligned with MAFFT 7.407 (Katoh and Standley 2013) using the ‘auto’ option. Alignments were trimmed with Gblocks 0.91 b (Castresana 2000) using relaxed settings and concatenated. Maximum likelihood analysis of the partitioned matrix was performed in RAxML 8.2.4 (Stamatakis 2014) using the GTR + GAMMA model with rapid bootstrapping. A cox1 tree including all publicly available scaphopod sequences was also reconstructed using this approach, but this failed to provide an unambiguous identification for Gadilida sp. (data not shown). Phylogenetic analysis of 15 mt genes recovered Scaphopoda, Dentaliida, and Gadilida monophyletic with maximal support (Figure 1).

**Figure 1.**
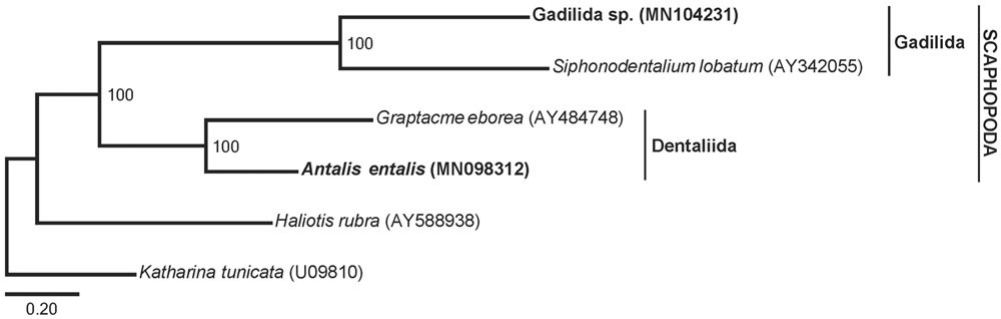
Maximum likelihood tree showing relationships among scaphopod species with sequenced mitochondrial genomes. Bootstrap support values are shown at each node. Scale bar indicates substitutions per site. Newly sequenced taxa are listed in bold.
